# Infection prevention and healthcare epidemiology professionals in low- and middle-income countries: a needs assessment survey and call for action

**DOI:** 10.1136/bmjgh-2024-018265

**Published:** 2025-12-19

**Authors:** Pranavi Sreeramoju, Xiaoyan Song, Ana Cecilia Bardossy, Jose Cadena, Corey A Forde, Payal Patel, Jorge Salinas, Catherine Tolliver, Bassem Zayed, Sarah L Krein

**Affiliations:** 1 Internal Medicine—Infectious Diseases, University of Pennsylvania Perelman School of Medicine, Philadelphia, Pennsylvania, USA; 2 Children’s National Hospital, Washington, DC, USA; 3 Centers for Disease Control and Prevention, Atlanta, Georgia, USA; 4 Department of Medicine, Infectious Diseases, UT Health San Antonio Long School of Medicine, San Antonio, Texas, USA; 5 Queen Elizabeth Hospital, Bridgetown, Barbados; 6 Division of Infectious Diseases, Intermountain Health, Salt Lake City, Utah, USA; 7 Stanford University School of Medicine, Palo Alto, California, USA; 8 The Society for Healthcare Epidemiology of America, Arlington, Virginia, USA; 9 WHO, Geneva, Switzerland; 10 University of Michigan, Ann Arbor, Michigan, USA

**Keywords:** Delivery of Health Care, Health Personnel, Global Health, Health systems, Public Health

## Abstract

**Introduction:**

More than 75% of the global population resides in low- and middle-income countries (LMICs), where healthcare-associated infection rates are notably higher than in high-income countries. Little is known about the professional experiences, perceptions and needs of infection prevention and healthcare epidemiology professionals (IPHEP) practising in these countries.

**Methods:**

A voluntary and anonymous online survey of IPHEP in LMICs was conducted via open invitations on social media and email from October 2022 to January 2023. The survey covered five domains: (1) Survey Responders, Practice Setting and Programme Characteristics; (2) Job Responsibilities, Training and Professional Development; (3) Workload and Work Environment; (4) COVID-19 Response; and (5) Priorities and Needs. Descriptive statistics were generated for the total sample and each World Bank region.

**Results:**

The number of survey respondents was 148, who represented 28/138 (20.3%) LMICs. They reported receiving formal training in infection prevention (80/94, 85.1%), antimicrobial stewardship (44/94, 46.8%), quality improvement and patient safety tools (55/94, 58.5%) and leadership (37/94, 39.4%). Importantly, 48.8% (42/86) reported job burnout. During the COVID-19 pandemic, 55/102 (53.9%) respondents reported their programme as effective or extremely effective, and 58/102 (56.9%) reported moderate or extreme financial hardship for their facility. Hand hygiene, improving antibiotic use and preventing multidrug-resistant organisms were ranked as top three priorities to be addressed, with specific resource needs identified for each programme by 89.0%, 95.0% and 93.8% of the survey respondents, respectively.

**Conclusion:**

This survey provides crucial insights into the realities faced by IPHEP in LMICs, emphasising the critical need for developing and strengthening workforce, supporting their organisational environments, allocating resources strategically for infection prevention and control initiatives, as well as improving their connectivity with other IPHEP colleagues across the world to foster greater collaboration and support.

**WHAT IS ALREADY KNOWN ON THIS TOPIC**

- More than 75% of the global population resides in low- and middle-income countries (LMICs), where healthcare-associated infection rates are notably higher than in high-income countries.
- Previous studies on the state of infection prevention and control (IPC) programmes did not capture the perceptions and needs of infection prevention and healthcare epidemiology personnel (IPHEP) in LMICs.

**WHAT THIS STUDY ADDS**

- This survey captured the viewpoints of IPHEP in LMICs related to their practice settings, job responsibilities, training gaps, workload challenges, work environment, COVID-19 response strategies and perceived priorities and needs.
- The study identified a substantial gap in formal training for job-specific responsibilities, with less than half of respondents indicating adequate training beyond basic infection prevention.
- The study also identified workload issues and challenging work environments.

**HOW THIS STUDY MIGHT AFFECT RESEARCH, PRACTICE OR POLICY**

- This study presents a call for action to address the need for IPHEP workforce development, supportive organisational environments and targeted resource allocation for IPC initiatives in LMICs.

## Introduction

 Infection prevention and healthcare epidemiology professionals (IPHEP) are responsible for ensuring safe healthcare environments and preventing the spread of infectious diseases within healthcare facilities all over the world.^1^ Over the decades, they have led infection prevention and control (IPC) programmes, developing evidence-based policies and procedures, conducting infectious disease surveillance, educating and training healthcare personnel (HCP) and responding to public health crises such as Ebola and COVID-19. The COVID-19 pandemic has further underscored the importance of IPC in healthcare facilities and community settings amidst rapidly evolving knowledge of pathophysiology of emerging pathogens and epidemiology of infectious diseases. These professionals work closely with administrators, clinicians and other frontline personnel in healthcare facilities, as well as with public health professionals in their communities. Increasingly, they also oversee antimicrobial stewardship (AS) programmes, provide input into diagnostic stewardship (DS) activities and provide occupational health recommendations for HCP exposed to infectious diseases. IPHEP practising in low- and middle-income countries (LMICs) where 75% of the world’s population resides^2^ are generally thought of as having resource challenges, although specific details are not known. We undertook this study with the goal of understanding the professional experiences, job responsibilities and training received, work environment and programme needs and priorities of IPHEP practising in LMICs.

Of the 218 economies categorised by the World Bank in 2021–2022, 27 (12.4%) were low-income countries (LIC), 55 (25.2%) lower middle-income countries (LMIC), 56 (25.7%) upper middle-income (UMIC) and 80 (36.7%) high-income countries (HIC).^2^ In HIC, IPC programmes are generally mandated to maintain safe and sanitary healthcare environments, staffed by qualified personnel with appropriate education, training and experience. Key competencies for infection preventionists (IPs) and healthcare epidemiologists (HEs) have been outlined by professional societies such as the Association for Professionals in Infection Control and Epidemiology (APIC) and the Society for Healthcare Epidemiology of America (SHEA).^3 4^

Globally, the WHO supports and coordinates IPC practices.^1^ A recent WHO survey highlighted significant variations in IPC programme structures and processes, particularly underscoring the critical need for robust IPC infrastructure worldwide.^5^ Specifically, IPC programmes are vastly different between HIC and LMICs, and these differences are reflected in programme outcomes, such as healthcare-associated infection (HAI) rates, which are higher in LMICs compared with HIC. Previous studies have also highlighted staffing challenges in LMICs.^6 7^ However, the professional experiences and needs of IPHEP in LMICs, which may be unique and distinct from those practising in HIC, and the perspectives of IPHEP leading and practising in IPC programmes in resource-limited countries remain largely unknown. This paper explores and summarises the perspectives of IPHEP in LMICs and provides a call for action. It targets IP, HE, healthcare administrators and professional societies in LMICs, as well as global healthcare advocacy groups.

## Methods

### Definitions

For the purposes of this paper, an HE is a physician, nurse or an individual with a doctoral degree or equivalent who has acquired specialty training in IPC practices and oversees an IPC programme. An IP is an individual, other than the HE, who manages and/or performs day-to-day activities within an IPC programme (with or without certification in infection control). Anyone practising infection prevention or healthcare epidemiology regardless of job title is referred to as an IPHEP. Depending on the context, an IPC programme in a facility may also be called an ‘infection prevention program’, ‘healthcare epidemiology’, ‘infection prevention’ or ‘infection control’. The word ‘country’ is used in place of the World Bank term ‘economy’ to enhance readability.

Although the World Bank categorises economies and not countries, we used the word countries in this paper for the sake of better readability. Income categories in 2021–2022 were used to categorise countries as LIC, LMIC, UMIC or HIC in this paper. The pluralised acronym LMICs (in contrast to LMIC used for lower middle income countries) is used when referring to the low- and middle-income countries as a broad category.^2^

### Study design and participants

An online electronic survey of IPHEP practising in LMICs was conducted over a 12-week period from October 2022 to January 2023. The survey tool was developed by the study team through discussion and consensus. It included questions from the WHO IPC assessment framework tool core component 1,^8^ as well as questions adapted from prior studies assessing hospital infection prevention practices.^9 10^ The survey covered five domains: (1) Survey Responders, Practice Setting and Programme Characteristics; (2) Job Responsibilities, Training and Professional Development; (3) Workload and Work Environment; (4) COVID-19 Response; and (5) Priorities and Needs. Participants had the option to provide free-text comments at the end of each domain. Prior to distribution, the survey instrument was pretested by members of the study team and several IPHEP colleagues. SHEA staff administered the survey using the Alchemer self-service survey software (Alchemer, Louisville, Colorado, USA). The platform includes preconfigured surveys, workflows and feedback collection tools, customisable surveys and reports. The survey link, QR code and information were disseminated via emails from SHEA to its international members and to the WHO Global IPC Network.^11^ Additionally, it was posted on social media platforms such as X and LinkedIn. The survey did not have a prespecified sampling target because of its exploratory and information gathering nature. The survey tool is included in the online supplemental file 1. Participation was voluntary, anonymous and confidential.

### Data management and analysis

Responses were included from all participants who identified the World Bank region where they practised. Data were aggregated and analysed by each of the six World Bank regions containing LMICs: East Asia and Pacific (EAP), Europe and Central Asia (ECA), Latin America and Caribbean (LAC), Middle East and North Africa (MENA), South Asia (SA) and sub-Saharan Africa (SSA). Descriptive frequencies were generated using the survey platform and are presented in this paper. Results from regions with fewer than 10 responses are not shown separately in the data tables but are included in the total results. Comparative analyses and significance testing were not conducted as they were not aligned with the primary purpose of this study.

## Results

**Figure 1:**
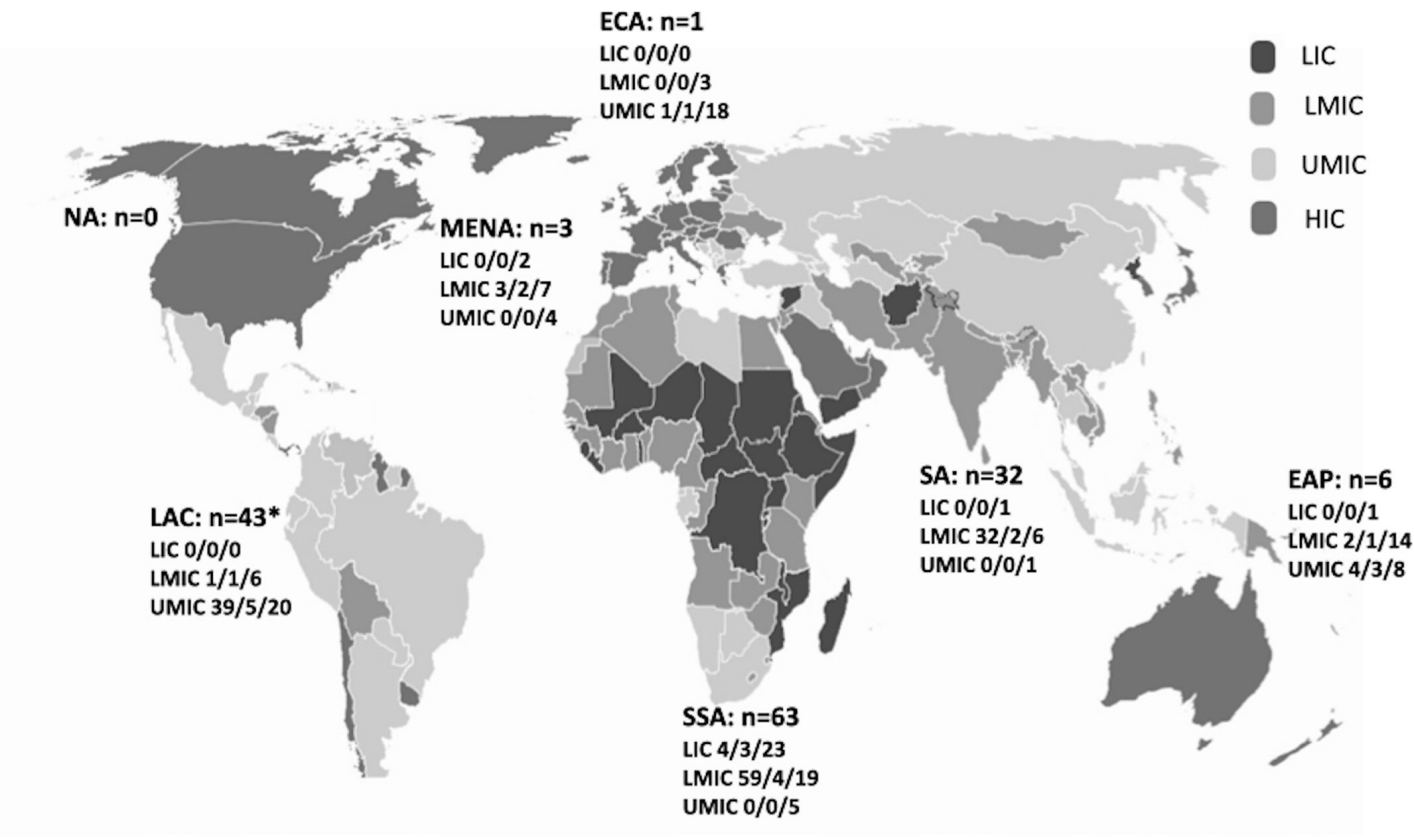
Representation of survey respondents by World Bank region and income category (n=148). n is the number of survey respondents in each region. The numbers for each category of countries are shown as the number of survey responses/number of countries represented in the survey/number of countries in the income category in the region. *Three respondents in LAC did not identify the country they practise in. Map adopted from https://datatopics.worldbank.org/world-development-indicators/the-world-by-income-and-region.html. EAP, East Asia and Pacific; ECA, Europe and Central Asia; HIC, high-income country; LAC, Latin America and Caribbean; LIC, low-income country; LMIC, low- and middle-income country; MENA, Middle East and North Africa; NA, North America; SA, South Asia; SSA, sub-Saharan Africa; UMIC, upper middle-income country.

A total of 148 IPHEP respondents responded to the survey. The number of responses from LIC, LMIC and UMIC was 4 (2.6%), 97 (66.9%) and 44 (30.3%) respectively; 3 reported only their region and not their country. Geographically, the respondents were from 28 different LMICs (28/138 20.3%). The distribution of these countries was LIC, 3/27 (11.1%); LMIC, 10/55 (18.2%), and UMIC, 15/56 (26.8%). Three respondents did not identify the country in which they practised. 63 (43%) respondents represented 7 out of 47 (14.9%) countries in SSA, 32 (22%) represented 2 out of 8 (25%) countries in SA and 40 (27%) represented 12 out of 26 (46.1%) countries in LAC. Responses also came from smaller numbers in other regions: one from ECA, six from EAP across four countries and three from the MENA across two countries, as depicted in figure 1.

Among the respondents, 82 individuals (55.4%) answered all applicable survey questions, while 66 (44.6%) answered some questions. Responses are reported as percentages of those who answered each specific question.

### Survey respondents, practice setting and programme characteristics

Of the 148 respondents who identified their job role, the majority (73.0%) identified as IP, while a smaller number (12.8%) identified as HE. 60% of respondents worked in academic or teaching-affiliated facilities, and 39.2% in community-based settings. Most respondents described their facilities as government-owned (61.5%), with smaller percentages being privately for-profit (20.9%) or not-for-profit (15.5%). 16 (10.8%) respondents reported that their facility had more than 1000 inpatient beds; 91.9% had an emergency department in their facility, and 68.2% reported that their facility had 11 or more intensive care beds. Detailed characteristics are provided in online supplemental table 1.

Regarding IPC programmes, 67.3% of respondents reported having programmes with clearly defined objectives. However, while 69.4% reported senior leadership support in high-level meetings, only 51.9% reported clear commitment from senior leadership with an allocated budget. AS programmes were reported in 69.4% of facilities, with 12.3% indicating tele-stewardship provision and 10.5% receiving tele-stewardship. DS activities included strategies to reduce inappropriate urine (62.5%) and blood culture testing (58.0%). Additional details are provided in online supplemental table 2.

### Job responsibilities, training and professional development

Among respondents specifying their professional backgrounds (n=94), diversity was evident. Respondent backgrounds included nursing (31.9%), clinical medicine (44.7%), microbiology (26.6%), public health (21.3%), healthcare administration (7.4%) and other fields (12.8%). Formal training was reported by 85.1% in IPC, 46.8% in AS, 58.5% in quality improvement (QI) and patient safety and 39.4% in leadership. Additional results are shown in table 1.

**Table 1:** Job training and resources provided

	Total			Latin America and Caribbean			South Asia			Sub-Saharan Africa		
	N	n	%	N	n	%	N	n	%	N	n	%
Professional background training	94			24			20			43		
Nursing		30	31.9		4	16.7		0	0.0		22	51.2
Clinical medicine		42	44.7		19	79.2		12	60.0		9	20.9
Microbiology		25	26.6		5	20.8		9	45.0		11	25.6
Public health		20	21.3		3	12.5		1	5.0		15	34.9
Healthcare administration		7	7.4		0	0.0		2	10.0		4	9.3
Other		12	12.8		4	16.7		1	5.0		5	11.6
Formal training received for job role in infection prevention	94			24		0.0	20		0.0	43		0.0
No formal training		14	14.9		4	16.7		8	40.0		2	4.7
WHO training		25	26.6		5	20.8		3	15.0		16	37.2
European society training		2	2.1		1	4.2		0	0.0		0	0.0
US SHEA training		10	10.6		4	16.7		2	10.0		0	0.0
Training offered by local public health or ministry of health		42	44.7		11	45.8		6	30.0		21	48.8
Infection control training offered by local organisations or training programmes		59	62.8		14	58.3		10	50.0		33	76.7
Received formal training in antimicrobial stewardship	94	44	46.8	24	10	41.7	20	9	45.0	43	22	51.2
Received formal training in quality improvement and patient safety tools	94	55	58.5	24	14	58.3	20	9	45.0	43	28	65.1
Received formal leadership training	94	37	39.4	24	5	20.8	20	6	30.0	43	24	55.8
Support for conference attendance and ongoing job-related training	94			24		0.0	20		0.0	43		0.0
Paid leave only		30	31.9		9	37.5		10	50.0		10	23.3
Sponsorship only		13	13.8		6	25.0		0	0.0		6	14.0
Paid leave and sponsorship		13	13.8		3	12.5		2	10.0		5	11.6
Neither paid leave nor sponsorship		38	40.4		6	25.0		8	40.0		22	51.2
Facility provides technology to conduct surveillance activities	94	53	56.4	24	17	70.8	20	18	90.0	43	13	30.2
If technology not provided, tools used for routine job activities	41			7			2			30		
Paper		15	36.6		1	14.3		1	50.0		13	43.3
Personal cell phone		17	41.5		2	28.6		1	50.0		13	43.3
Availability of free Wi-Fi access at work	94	47	50.0	24	14	58.3	20	14	70.0	43	13	30.2
Use of own Wi-Fi to perform any work activities	47	40	85.1	10	8	80.0	6	5	83.3	30	27	90.0

N represents available answers for each of the questions.

SHEA, Society for Healthcare Epidemiology of America.

The respondents also carried out a variety of job responsibilities such as providing education to staff (68/84, 81%), developing and implementing policies and procedures (66/76, 86.8%), conducting surveillance to promptly identify and interrupt clusters and outbreaks (66/73, 90.4%) and serving as content experts to troubleshoot IPC related questions and concerns (65/73, 89.0%). However, the percentage who reported being responsible for a specific job, relative to the percentage who reported receiving job-specific training, varied. For example, while 86.6% reported ‘Serve as liaison between external public health agencies and the hospital’ as a job responsibility, only 32.8% received formal training specific to that job. On the other hand, 81.0% of respondents identified ‘Provide education to staff’ as a job responsibility and 61.9% reported the receipt of formal training specific to this responsibility. Detailed results are shown in table 2.

**Table 2:** Match between job responsibility and receipt of specific training

	Total					Latin America and Caribbean					South Asia					Sub-Saharan Africa				
	N	Job responsibility (n,%)		Formal training received (n, %)		N	Job responsibility (n, %)		Formal training received (n, %)		N	Job responsibility (n, %)		Formal training received (n, %)		N	Job responsibility (n, %)		Formal training received (n, %)	
Job responsibility																				
Provide education to staff	84	68	81.0	52	61.9	18	16	88.9	5	27.8	16	15	93.8	10	62.5	43	31	72.1	33	76.7
Develop and implement policies and procedures	76	66	86.8	30	39.5	21	19	90.5	5	23.8	13	13	100.0	4	30.8	35	27	77.1	20	57.1
Conduct surveillance per regulatory requirement	70	59	84.3	37	52.9	16	14	87.5	6	37.5	11	11	100.0	5	45.5	36	28	77.8	21	58.3
Compile and report infectious diseases to local public health authorities	68	60	88.2	31	45.6	20	20	100.0	7	35.0	13	13	100.0	5	38.5	29	21	72.4	16	55.2
Conduct surveillance to promptly identify and interrupt clusters and outbreaks	73	66	90.4	36	49.3	20	20	100.0	6	30.0	13	13	100.0	7	53.8	33	27	81.8	17	51.5
Serve as liaison between external public health agencies and the hospital	67	58	86.6	22	32.8	19	17	89.5	3	15.8	12	12	100.0	4	33.3	30	23	76.7	14	46.7
Serve as content expert to troubleshoot infection control related questions and concerns	73	65	89.0	35	47.9	21	20	95.2	7	33.3	12	12	100.0	5	41.7	33	26	78.8	19	57.6
Occupational health	56	46	82.1	24	42.9	10	6	60.0	4	40.0	11	11	100.0	5	45.5	30	24	80.0	13	43.3
Antimicrobial stewardship	70	60	85.7	31	44.3	17	16	94.1	5	29.4	14	14	100.0	6	42.9	33	25	75.8	17	51.5
Emerging infectious disease and bioterrorism preparedness and response	60	50	83.3	25	41.7	16	13	81.3	5	31.3	10	10	100.0	3	30.0	28	22	78.6	14	50.0
Implement bundles of care to reduce healthcare-associated infections	74	64	86.5	40	54.1	18	16	88.9	7	38.9	15	15	100.0	7	46.7	34	27	79.4	21	61.8

N represents available answers for each of the questions.

### Workload and work environment

When asked about their work experience, 42 of 86 respondents (48.8%) agreed or strongly agreed with the statement ‘I feel burned out from my work’. Write-in comments suggested heavy workloads and staff shortages were a commonly perceived source of burnout as illustrated by one respondent who wrote: ‘The workload is much as we don’t have enough staff’. In response to questions about their work environment, only 17/85 (20.0%) agreed or strongly agreed with the statement ‘In this facility, employees are expected to question leadership’, while 51/85 (60.0%) agreed or strongly agreed that ‘In this facility, authority is concentrated at the top’. Approximately one-quarter of respondents (22/85, 25.9%) agreed or strongly agreed with the statement ‘If you make a mistake at this facility, it is often held against you’. However, 49/85 (57.6%) responded never when asked whether they feel any pressure to NOT report HAIs at own facility, and most respondents (n=67/85, 78.8%) agreed or strongly agreed with the statement ‘When a medical error occurs at this facility, employees are encouraged to discuss mistakes in order to learn how to prevent similar future errors’. Additional results are shown in table 3.

**Table 3:** Workload and work environment

	Total			Latin America and Caribbean			South Asia			Sub-Saharan Africa		
	N	n	%	N	n	%	N	n	%	N	n	%
Work experience of the survey respondent: agree or strongly agree												
‘I feel burned out from my work’.	86	42	48.8	21	11	52.4	19	5	26.3	39	23	59.0
‘I have become more callous towards people since I took my job’.	85	17	20.0	20	6	30.0	19	3	15.8	39	6	15.4
‘Spiritual well-being is important for one’s emotional well-being’.	85	75	88.2	20	18	90.0	19	18	94.7	39	34	87.2
‘Religious or spiritual beliefs act as a source of comfort and strength during life’s ups and downs’.	85	70	82.4	20	13	65.0	19	16	84.2	39	35	89.7
‘An organized religious or spiritual community is important to me’.	85	58	68.2	20	9	45.0	19	9	47.4	39	36	92.3
‘Individual self-care practices are important to me’.	85	71	83.5	20	18	90.0	19	17	89.5	39	31	79.5
Respondent’s views of their workplace: agree or strongly agree												
‘I assert my views on important issues, even though my supervisor may disagree’.	85	70	82.4	20	18	90.0	19	18	94.7	39	30	76.9
‘I personally feel comfortable speaking up when I see a physician not clean his or her hands’.	85	61	71.8	20	12	60.0	19	15	78.9	39	30	76.9
‘When a medical error occurs at this facility, employees are encouraged to discuss mistakes in order to learn how to prevent similar future errors’.	85	67	78.8	20	14	70.0	19	16	84.2	39	31	79.5
‘Leadership is driving us to be a safety-centered institution’.	85	65	76.5	20	12	60.0	19	16	84.2	39	31	79.5
‘I would feel safe being treated here as a patient’.	85	57	67.1	20	13	65.0	19	15	78.9	39	25	64.1
‘If you make a mistake at this facility, it is often held against you’.	85	22	25.9	20	6	30.0	19	6	31.6	39	10	25.6
‘Employees at this facility are able to bring up problems and tough issues’.	85	51	60.0	20	9	45.0	19	16	84.2	39	21	53.8
‘It is safe to try something new at this facility’.	85	60	70.6	20	11	55.0	19	17	89.5	39	28	71.8
‘At this facility, people are too busy to invest time in improvement’.	85	34	40.0	20	7	35.0	19	8	42.1	39	18	46.2
‘In this facility, employees are expected to question leadership’.	85	17	20.0	20	3	15.0	19	3	15.8	39	9	23.1
‘In this facility, authority is concentrated at the top’.	85	51	60.0	20	13	65.0	19	11	57.9	39	22	56.4
Feeling any pressure to NOT report healthcare-associated infections at own facility	85			20		0.0	19			39		
Always	85	2	2.4	20	2	10.0	19	0	0.0	39	0	0.0
Sometimes	85	18	21.2	20	3	15.0	19	3	15.8	39	9	23.1
Rarely	85	16	18.8	20	3	15.0	19	2	10.5	39	11	28.2
Never	85	49	57.6	20	12	60.0	19	14	73.7	39	19	48.7
Importance of hand hygiene among all patient safety issues at own facility: very or extremely important	85	71	83.5	20	16	80.0	19	17	89.5	39	33	84.6
Agreement (agree or strongly agree) with ‘I feel safe carrying out my work role during the COVID-19 pandemic’.	85	59	69.4	20	13	65.0	19	17	89.5	39	26	66.7

N represents available answers for each of the questions.

### COVID-19 response

Overall, 55 out of 102 respondents (53.9%) reported that their COVID-19 response was very or extremely effective. The majority, 81/102 (79.4%), reported relying on the national ministry of health in their own country for information and guidance during the pandemic. Approximately two-thirds, 67/102 (65.7%) relied on guidance from the WHO, and fewer relied on guidance from the US Centers for Disease Control and Prevention (CDC) (55.9%), Infectious Diseases Society of America (IDSA) (17.6%) and SHEA (17.6%). 88 (86.3%) reported that their facility experienced staff shortages due to absences and/or illness, and 41 (40.2%) reported that their facility experienced increased loss of staff due to resignations. 58 (56.9%) reported that their facility experienced moderate or extreme financial hardship. Complete results of this section are reported in online supplemental table 3.

### Programmatic priorities and needs

Hand hygiene (91.3%) was overwhelmingly prioritised among the top three priorities based on responses from 81 survey respondents, followed by improving antibiotic use (46.9%) and multidrug-resistant organism (MDRO) control (45.7%). Fewer respondents ranked other areas among the top three priorities: preventing surgical site infections, 42.0%; outbreak control and prevention, 28.4%; preventing central line-associated bloodstream infections, 19.7%; responding to emerging infectious diseases, 18.5%; preventing ventilator-associated events and pneumonia, 11.1%.

When surveyed on their perception of programme needs, the respondents reported having several needs. These needs ranged from additional training for themselves or for their team members, additional supplies, staff or lab capacity to non-material needs such as greater hospital leadership support and engagement. A majority of respondents identified one or more needs related to hand hygiene, 89.0%; preventing MDRO, 93.8%; improving antibiotic use, 95.0%; preventing central line-associated bloodstream infections, 87.6%; preventing surgical site infections, 86.4%; preventing ventilator-associated events or ventilator-associated pneumonia, 82.7%; outbreak identification and management, 86.4%; and, emerging infectious disease preparation and response, 90%. Complete survey results related to perceived needs are reported in table 4 for the three priority areas, and online supplemental table 4 for other important areas.

**Table 4:** Perceived resource needs for high priority functions

	Total			Latin America and Caribbean			South Asia			Sub-Saharan Africa		
	N	n	%	N	n	%	N	n	%	N	n	%
Hand hygiene	82			20			18			37		
Not applicable: I am not responsible for this activity		1	1.2		1	5.0		0	0.0		0	0.0
No additional resources needed		8	9.8		2	10.0		4	22.2		1	2.7
I need additional training		4	4.9		0	0.0		1	5.6		3	8.1
My team members need additional training		8	9.8		2	10.0		3	16.7		3	8.1
I need additional resources to educate frontline clinical personnel		18	22.0		5	25.0		3	16.7		8	21.6
I need additional hospital leadership support and engagement		26	31.7		6	30.0		2	11.1		15	40.5
I need additional supplies		11	13.4		1	5.0		3	16.7		7	18.9
I need additional IPC staff		6	7.3		3	15.0		2	11.1		0	0.0
I need additional lab capacity		0	0.0		0	0.0		0	0.0		0	0.0
Preventing MDRO	81			19			18			37		
Not applicable: I am not responsible for this activity		0	0.0		0	0.0		0	0.0		0	0.0
No additional resources needed		5	6.2		1	5.3		2	11.1		1	2.7
I need additional training		16	19.8		2	10.5		3	16.7		11	29.7
My team members need additional training		10	12.3		1	5.3		5	27.8		4	10.8
I need additional resources to educate frontline clinical personnel		23	28.4		4	21.1		6	33.3		10	27.0
I need additional hospital leadership support and engagement		13	16.0		7	36.8		0	0.0		3	8.1
I need additional supplies		1	1.2		0	0.0		1	5.6		0	0.0
I need additional IPC staff		3	3.7		1	5.3		1	5.6		1	2.7
I need additional lab capacity		10	12.3		3	15.8		0	0.0		7	18.9
Improving antibiotic use	80			19			17			37		
Not applicable: I am not responsible for this activity		1	1.3		0	0.0		0	0.0		1	2.7
No additional resources needed		3	3.8		1	5.3		0	0.0		1	2.7
I need additional training		11	13.8		3	15.8		2	11.8		5	13.5
My team members need additional training		15	18.8		3	15.8		5	29.4		6	16.2
I need additional resources to educate frontline clinical personnel		30	37.5		6	31.6		7	41.2		16	43.2
I need additional hospital leadership support and engagement		17	21.3		6	31.6		3	17.6		5	13.5
I need additional supplies		0	0.0		0	0.0		0	0.0		0	0.0
I need additional IPC staff		0	0.0		0	0.0		0	0.0		0	0.0
I need additional lab capacity		3	3.8		0	0.0		0	0.0		3	8.1

N represents available answers for each of the questions.

IPC, infection prevention and control; MDRO, multidrug-resistant organisms.

## Discussion

This survey captured the perspectives of IPHEP practising in LMICs through targeted outreach via social media and professional organisation networks. With responses from 148 IPHEP representatives representing a fifth of LMICs, including a substantial number of responses from three World Bank regions, SA, SSA and LAC, the survey provides valuable insights into various facets including practice settings and IPC programme characteristics, job responsibilities and training, workload and work environment, COVID-19 response and perceived priorities and needs.

The first key survey finding is that IPHEP practitioners in LMICs reported substantial gaps in formal training for specific job responsibilities. Even though the majority, 85.1%, reported receiving formal training in IPC, less than two-thirds reported receiving formal training in QI and patient safety, AS and leadership. The IPHEP also reported insufficient training for several job responsibilities, which should be of global concern. A well-trained and skilled workforce is critical for success with global IPC, AS and pandemic response. To bridge the training gaps, we can encourage the IPHEP in LMIC to use several educational resources offered online, with the majority being free to users and some requiring registration. Specific to infection prevention guidance for COVID-19, the majority of the survey respondents reported relying on guidance from the WHO, with CDC as a close second, and far fewer reporting use of guidance from US-based professional societies. These results might be due to three reasons: (1) WHO guidance and some CDC guidance are available in English as well as other major languages; (2) WHO guidance, and some CDC resources are specifically created to address needs in LMICs; and (3) the US-based professional societies may not be well known in the countries where the survey respondents practise.

Some of the key education and training resources available to everyone, including IPHEP in LMICs, are listed below.^12^,^19^ While they are not a substitute for in-person training, they can mitigate some of the training needs of this important segment of the healthcare workforce.

- *Open WHO platform:* offers free courses on antimicrobial resistance, IPC, tuberculosis, outbreak preparedness, pandemic readiness and clinical management of emerging infections in multiple languages. Registration may be required for certain content.
- *Pan-American Health Organization:* endorsed by WHO, provides resources in various languages on preventing communicable and non-communicable diseases, including drug-resistant organisms. Some content requires registration.
- *CDC:* offers comprehensive information on HAIs, MDRO, prevention strategies, statistics, research and public reporting.
- *SHEA:* provides a compendium for preventing HAI, updated regularly and offers free educational modules on HAI prevention, AS, high-consequence pathogens and podcasts.
- *IDSA:* offers guidelines for clinical management, AS, public health policy updates and educational resources, including free features and specialised courses like the ‘IDSA Antimicrobial Stewardship Curriculum for ID Fellows’.
- *APIC:* provides educational resources including training, textbooks and tools for certification in IPC.
- *Joint Commission International:* sets quality standards, certifies healthcare facilities and provides resources on infection prevention, AS and patient safety. Offers a mix of free and paid resources, widely used internationally.
- *Open access journals:* examples include ‘Antimicrobial Stewardship & Healthcare Epidemiology’ (SHEA), ‘Infection Prevention in Practice’ (Healthcare Infection Society) and ‘International Journal of Infection Control’, providing global access to high-quality research in IPC and AS.
- *QI, patient safety and leadership training:* various free resources are available for enhancing QI, safety practices and leadership skills in healthcare settings from organisations such as the Institute for Healthcare Improvement, Agency for Healthcare Research and Quality and others.

Another finding related to training is that healthcare epidemiology as a field is less well developed in LMICs as compared with HIC. As articulated by a respondent: ‘In my country, IPC is dominated by nurses with basic training, while medical officers lack clear career pathways’. Lack of clear career pathways for physicians may be a disincentive for those interested in entering the field. Although some LMICs have facilities in which physicians lead IPC programmes and teams, a shortage of physicians in this professional space could also be a reflection on resource constraints that potentially limit a facility’s ability to pay physicians to oversee IPC programmes. As physicians have an important role to play in the IP and HE space, healthcare leaders and professional societies in LMICs may need to engage in specific advocacy and make investments to attract and retain them.

The second key survey finding is the need for greater senior leadership involvement in improving the workload and work environments. The survey revealed positive support for IPC programmes from senior leadership and clinical staff across many facilities, indicating organisational commitment to patient and staff safety despite resource constraints. Nonetheless, workload and work environment challenges were prevalent, with nearly half of respondents reporting burnout attributed to heavy workloads and staff shortages. The survey also suggests the presence of a high power distance in the work environments, with only 20% agreeing or strongly agreeing that at their facility employees are expected to question leadership and 60% indicating that authority is concentrated at the top.^20^ Despite this, there was a perceived high degree of psychological safety among respondents, which is crucial in facilitating effective IPC practices and mitigating HAIs. Strengthening safety cultures and leadership structures within healthcare facilities, alongside advocacy for IPC resource allocation, is essential steps toward enhancing IPC practices in LMICs.^21 22^ Strategies to reduce burnout and enhance workforce well-being, including supportive organisational climates and self-care practices, are also imperative in sustaining a resilient IP and HE workforce in LMICs.^23 24^

The third key finding is the clear need for infrastructure development. The majority of IPHEP reported having additional needs to carry out their job functions. Perceived needs and priorities highlighted critical challenges in IPC implementation in LMICs, including resource limitations and competing priorities. Addressing these challenges requires targeted investments in IPC training, professional development and infrastructure, emphasising hand hygiene, AS and MDRO control as top priorities.^25^,^28^ Only half of the respondents reported the presence of AS programmes in their facilities. Given the critical role of AS in curbing inappropriate antibiotic use, particularly amidst low availability of DS strategies, there is a clear need for infrastructure development, skill enhancement and leadership support in LMICs. The COVID-19 pandemic also significantly impacted IPC practices globally. While respondents generally perceived their facility’s COVID-19 response positively, the interpretation of these findings is tempered by regional variations in community transmission rates and mitigation measure compliance. The COVID-19 pandemic underscored the need for robust IPC strategies and resource resilience, evident in increased workload and supply shortages reported globally.^29^,^32^

### Limitations

Notable limitations to this survey include the nature of outreach via social media and emailing survey link, administration of the survey in English although translation within app or browser was allowed, low number of respondents and lack of representation from three of the six World Bank regions with LMICs. Despite aggressive social media and email outreach, IPHEP in only 20.3% of the LMICs could be reached, with elicitation of a sufficient number of responses from only half the World Bank regions. The reach within LIC was the lowest, and the reach within UMIC was the highest, suggesting the presence of many facilitators to participate in a survey such as this among this latter group. Dissemination of the survey via SHEA might be a reason for lower reach to the IPHEP in LIC. The survey respondents may also be among the better trained IPHEP compared with those who did not answer the survey for the same reason. We were unable to avoid duplicate survey responses from similar hospitals as the logistics of such an undertaking were too complicated and challenging. Consequently, our analysis was conducted at the individual respondent level.

## Conclusions

In conclusion, this survey offers valuable insights into the realities faced by IPHEP in LMICs, emphasising the importance of workforce development, supportive organisational environments, targeted resource allocation for IPC initiatives and improving their connectivity with colleagues across the world. Data reported in this study are crucial for shaping global initiatives aimed at enhancing IPC infrastructures tailored to LMICs context. Collaborative efforts involving educational institutions and health authorities are needed to embed standardised IPC curricula into healthcare education programmes, ensuring preparedness and competence among future IPC professionals. Continued dissemination of available resources and sustained investments in IPC will be pivotal in fortifying global health security against emerging infections and future pandemics.

## Supplementary material

10.1136/bmjgh-2024-018265 online supplemental table 1

10.1136/bmjgh-2024-018265 online supplemental file 1

## Data Availability

All data relevant to the study are included in the article or uploaded as supplementary information.
