# Supplementary material for: Infection prevention and healthcare epidemiology professionals in low- and middle-income countries: a needs assessment survey and call for action
Source: BMJ Glob Health. 2025 Dec 19;10(12):e018265. doi: 10.1136/bmjgh-2024-018265 (PMC12716524; doi:10.1136/bmjgh-2024-018265)
Supplement: online supplemental table 1 [file bmjgh-10-12-s001.docx]

**Infection Prevention and Healthcare Epidemiology Professionals in Low- and Middle- Income Countries: A Needs Assessment Survey and Call for Action**

Supplement Table 1: Survey Responders and Settings Where They Work

|  | **Total** | | |  | **Latin America & Caribbean** | | |  | **South Asia** | | |  | **Sub-Saharan Africa** | | |
| --- | --- | --- | --- | --- | --- | --- | --- | --- | --- | --- | --- | --- | --- | --- | --- |
|  | N | n | % |  | N | n | % |  | N | n | % |  | N | n | % |
| Completeness of Survey Submitted | 148 |  |  |  |  |  |  |  |  |  |  |  |  |  |  |
| Complete |  | 82 | 55.0% |  |  | 20 | 46.5% |  |  | 18 | 56.3% |  |  | 37 | 57.8% |
| Partial |  | 66 | 45.0% |  |  | 23 | 53.5% |  |  | 14 | 43.8% |  |  | 27 | 42.2% |
| Role | 148 |  |  |  | 43 |  |  |  | 32 |  |  |  | 63 |  |  |
| Healthcare Epidemiologist |  | 19 | 12.8% |  |  | 10 | 23.3% |  |  | 4 | 12.5% |  |  | 2 | 3.2% |
| Infection Preventionist |  | 108 | 73.0% |  |  | 28 | 65.1% |  |  | 22 | 68.8% |  |  | 52 | 82.5% |
| Work Setting | 148 |  |  |  | 43 |  |  |  | 32 |  |  |  | 63 |  |  |
| Community-based |  | 58 | 39.2% |  |  | 21 | 48.8% |  |  | 10 | 31.3% |  |  | 23 | 36.5% |
| Academic/ Teaching Affiliated |  | 90 | 60.8% |  |  | 22 | 51.2% |  |  | 22 | 68.8% |  |  | 40 | 63.5% |
|  | 148 |  |  |  | 43 |  |  |  | 32 |  |  |  | 63 |  |  |
| Primary care only |  | 6 | 4.1% |  |  | 1 | 2.3% |  |  | 2 | 6.3% |  |  | 3 | 4.8% |
| Acute care, tertiary hospital |  | 103 | 69.6% |  |  | 32 | 74.4% |  |  | 22 | 68.8% |  |  | 42 | 66.7% |
| Long-term care |  | 11 | 7.4% |  |  | 0 | 0.0% |  |  | 3 | 9.4% |  |  | 8 | 12.7% |
| Specialty |  | 13 | 8.8% |  |  | 5 | 11.6% |  |  | 5 | 15.6% |  |  | 3 | 4.8% |
|  | 148 |  |  |  | 43 |  |  |  | 32 |  |  |  | 63 |  |  |
| Government-owned |  | 91 | 61.5% |  |  | 16 | 37.2% |  |  | 12 | 37.5% |  |  | 55 | 87.3% |
| Privately owned, not for profit |  | 23 | 15.5% |  |  | 14 | 32.6% |  |  | 2 | 6.3% |  |  | 6 | 9.5% |
| Privately owned, for profit |  | 31 | 20.9% |  |  | 11 | 25.6% |  |  | 18 | 56.3% |  |  | 1 | 1.6% |
| Number of inpatient beds | 148 |  |  |  | 43 |  |  |  | 32 |  |  |  | 63 |  |  |
| None |  | 10 | 6.8% |  |  | 3 | 7.0% |  |  | 0 | 0.0% |  |  | 4 | 6.3% |
| <200 |  | 31 | 20.9% |  |  | 19 | 44.2% |  |  | 7 | 21.9% |  |  | 5 | 7.9% |
| 200-500 |  | 48 | 32.4% |  |  | 12 | 27.9% |  |  | 7 | 21.9% |  |  | 29 | 46.0% |
| 500-1000 |  | 43 | 29.1% |  |  | 7 | 16.3% |  |  | 10 | 31.3% |  |  | 23 | 36.5% |
| >1000 |  | 16 | 10.8% |  |  | 2 | 4.7% |  |  | 8 | 25.0% |  |  | 2 | 3.2% |
| Number of ICU beds | 148 |  |  |  | 43 |  |  |  | 32 |  |  |  | 63 |  |  |
| None |  | 19 | 12.8% |  |  | 6 | 14.0% |  |  | 2 | 6.3% |  |  | 10 | 15.9% |
| <11 |  | 28 | 18.9% |  |  | 8 | 18.6% |  |  | 2 | 6.3% |  |  | 16 | 25.4% |
| 11-20 |  | 46 | 31.1% |  |  | 10 | 23.3% |  |  | 4 | 12.5% |  |  | 31 | 49.2% |
| 21-30 |  | 12 | 8.1% |  |  | 6 | 14.0% |  |  | 2 | 6.3% |  |  | 3 | 4.8% |
| 31-40 |  | 16 | 10.8% |  |  | 9 | 20.9% |  |  | 3 | 9.4% |  |  | 2 | 3.2% |
| >41 |  | 27 | 18.2% |  |  | 4 | 9.3% |  |  | 19 | 59.4% |  |  | 1 | 1.6% |
| Specialty Services Offered | 148 |  |  |  | 43 |  |  |  | 32 |  |  |  | 63 |  |  |
| Outpatient dialysis |  | 106 | 71.6% |  |  | 25 | 58.1% |  |  | 26 | 81.3% |  |  | 47 | 74.6% |
| Blood and marrow transplant |  | 41 | 27.7% |  |  | 16 | 37.2% |  |  | 14 | 43.8% |  |  | 8 | 12.7% |
| Solid-organ transplant |  | 45 | 30.4% |  |  | 14 | 32.6% |  |  | 21 | 65.6% |  |  | 6 | 9.5% |
| Cardiovascular surgery |  | 81 | 54.7% |  |  | 25 | 58.1% |  |  | 23 | 71.9% |  |  | 27 | 42.9% |
| Newborn |  | 111 | 75.0% |  |  | 29 | 67.4% |  |  | 22 | 68.8% |  |  | 53 | 84.1% |
| Number of Outpatient facilities | 148 |  |  |  | 43 |  |  |  | 32 |  |  |  | 63 |  |  |
| 0 |  | 9 | 6.1% |  |  | 7 | 16.3% |  |  | 1 | 3.1% |  |  | 1 | 1.6% |
| 1-5 |  | 31 | 20.9% |  |  | 16 | 37.2% |  |  | 5 | 15.6% |  |  | 6 | 9.5% |
| 6-10 |  | 26 | 17.6% |  |  | 6 | 14.0% |  |  | 1 | 3.1% |  |  | 19 | 30.2% |
| 11-20 |  | 29 | 19.6% |  |  | 4 | 9.3% |  |  | 4 | 12.5% |  |  | 18 | 28.6% |
| >20 |  | 53 | 35.8% |  |  | 10 | 23.3% |  |  | 21 | 65.6% |  |  | 19 | 30.2% |
| Facility has Emergency Department | 148 | 136 | 91.9% |  | 43 | 40 | 93.0% |  | 32 | 28 | 87.5% |  | 63 | 59 | 93.7% |
| SHEA member | 82 | 26 | 31.7% |  | 20 | 7 | 35.0% |  | 18 | 8 | 44.4% |  | 37 | 5 | 13.5% |
| Language translation app used to complete survey | 82 | 3 | 3.7% |  | 20 | 2 | 10.0% |  | 18 | 0 | 0.0% |  | 37 | 1 | 2.7% |

N represents available answers for each of the questions.

Supplement Table 2: Characteristics of Infection Prevention and Control, and Antimicrobial Stewardship Programs

|  | **Total** | | |  | **Latin America & Caribbean** | | |  | **South Asia** | | |  | **Sub-Saharan Africa** | | |
| --- | --- | --- | --- | --- | --- | --- | --- | --- | --- | --- | --- | --- | --- | --- | --- |
|  | N | n | % |  | N | n | % |  | N | n | % |  | N | n | % |
| IPC program present | 113 |  |  |  | 30 |  |  |  | 25 |  |  |  | 51 |  |  |
| No |  | 5 | 4.4% |  |  | 3 | 10.0% |  |  | 1 | 4.0% |  |  | 1 | 2.0% |
| Yes, without clear objectives |  | 32 | 28.3% |  |  | 8 | 26.7% |  |  | 5 | 20.0% |  |  | 17 | 33.3% |
| Yes, with clear objectives |  | 76 | 67.3% |  |  | 19 | 63.3% |  |  | 19 | 76.0% |  |  | 33 | 64.7% |
| If IPC program present, | 108 |  |  |  | 27 |  |  |  | 24 |  |  |  | 50 |  |  |
| Respondent is in charge of IPC program |  | 76 | 70.4% |  |  | 21 | 77.8% |  |  | 15 | 62.5% |  |  | 34 | 68.0% |
| Respondent is not in charge of IPC program |  | 32 | 29.6% |  |  | 6 | 22.2% |  |  | 9 | 37.5% |  |  | 16 | 32.0% |
| IPC program is supported by an IPC team |  | 92 | 85.2% |  |  | 21 | 77.8% |  |  | 22 | 91.7% |  |  | 44 | 88.0% |
| IPC program is not supported by an IPC team |  | 2 | 1.9% |  |  | 2 | 7.4% |  |  | 0 | 0.0% |  |  | 0 | 0.0% |
| There is only an IPC focal person |  | 14 | 13.0% |  |  | 4 | 14.8% |  |  | 2 | 8.3% |  |  | 6 | 12.0% |
| The team has at least one full-time IPC professional per 250 or more beds |  | 30 | 27.8% |  |  | 7 | 25.9% |  |  | 8 | 33.3% |  |  | 14 | 28.0% |
| The team has at least one full-time IPC professional per less than 250 beds |  | 43 | 39.8% |  |  | 14 | 51.9% |  |  | 12 | 50.0% |  |  | 11 | 22.0% |
| The IPC team has only a part-time IPC professional |  | 25 | 23.1% |  |  | 6 | 22.2% |  |  | 1 | 4.2% |  |  | 18 | 36.0% |
| The IPC team does not have an IPC professional |  | 7 | 6.5% |  |  | 0 | 0.0% |  |  | 1 | 4.2% |  |  | 6 | 12.0% |
| Respondent does not know if the program has at least one full-time IPC professional |  | 3 | 2.8% |  |  | 0 | 0.0% |  |  | 2 | 8.3% |  |  | 1 | 2.0% |
| IPC team composition | 92 |  |  |  | 21 |  |  |  | 22 |  |  |  | 44 |  |  |
| The IPC team includes both doctors and nurses |  | 91 | 98.9% |  |  | 21 | 100.0% |  |  | 22 | 100.0% |  |  | 43 | 97.7% |
| The IPC team does not include both doctors and nurses |  | 1 | 1.1% |  |  | 0 | 0.0% |  |  | 0 | 0.0% |  |  | 1 | 2.3% |
| Respondent does not know if the program has both doctors and nurses |  | 0 | 0.0% |  |  | 0 | 0.0% |  |  | 0 | 0.0% |  |  | 0 | 0.0% |
| The facility has an IPC committee actively supporting the IPC program | 108 | 94 | 87.0% |  | 27 | 24 | 88.9% |  | 24 | 23 | 95.8% |  | 50 | 42 | 84.0% |
| The facility does not have an IPC committee actively supporting the IPC program |  | 14 | 12.4% |  |  | 3 | 11.1% |  |  | 1 | 4.2% |  |  | 8 | 16.0% |
| Professional groups actively supporting the respondent's job role | 114 |  | 0.0% |  | 30 |  |  |  | 25 |  |  |  | 52 |  |  |
| Senior facility leadership |  | 81 | 71.7% |  |  | 17 | 56.7% |  |  | 20 | 80.0% |  |  | 39 | 75.0% |
| Senior clinical staff |  | 84 | 74.3% |  |  | 18 | 60.0% |  |  | 20 | 80.0% |  |  | 40 | 76.9% |
| Facility management |  | 75 | 66.4% |  |  | 13 | 43.3% |  |  | 18 | 72.0% |  |  | 39 | 75.0% |
| Whether job effectiveness measured | 113 |  | 0.0% |  | 30 |  | 0.0% |  | 25 |  | 0.0% |  | 51 |  |  |
| Respondent's job effectiveness is measured by clearly defined IPC objectives only |  | 26 | 23.0% |  |  | 6 | 20.0% |  |  | 4 | 16.0% |  |  | 14 | 27.5% |
| Respondent's job effectiveness is measured by clearly defined IPC objectives, and measurable outcome indicators |  | 30 | 26.5% |  |  | 9 | 30.0% |  |  | 4 | 16.0% |  |  | 16 | 31.4% |
| Respondent's job effectiveness is measured by clearly defined IPC objectives, measurable outcome indicators, and set future targets |  | 39 | 34.5% |  |  | 8 | 26.7% |  |  | 15 | 60.0% |  |  | 12 | 23.5% |
| Respondent's job effectiveness is not measured by clearly defined IPC objectives |  | 18 | 15.9% |  |  | 7 | 23.3% |  |  | 2 | 8.0% |  |  | 9 | 17.6% |
| Senior leadership show clear commitment with allocated budget | 108 | 56 | 51.9% |  | 27 | 15 | 55.6% |  | 24 | 18 | 75.0% |  | 50 | 18 | 36.0% |
| Senior leadership show clear support in high-level meetings | 108 | 75 | 69.4% |  | 27 | 18 | 66.7% |  | 24 | 22 | 91.7% |  | 50 | 30 | 60.0% |
| Antimicrobial Stewardship program present | 114 | 74 | 64.9% |  | 30 | 18 | 60.0% |  | 25 | 21 | 84.0% |  | 52 | 29 | 55.8% |
| The antimicrobial stewardship team has | 77 |  |  |  | 18 |  |  |  | 21 |  |  |  | 32 |  |  |
| Infection preventionist |  | 49 | 63.6% |  |  | 7 | 38.9% |  |  | 14 | 66.7% |  |  | 23 | 71.9% |
| ID physician |  | 53 | 68.8% |  |  | 15 | 83.3% |  |  | 14 | 66.7% |  |  | 20 | 62.5% |
| Hospital medicine or Internal medicine physician |  | 36 | 46.8% |  |  | 2 | 11.1% |  |  | 11 | 52.4% |  |  | 20 | 62.5% |
| Other physician |  | 26 | 33.8% |  |  | 2 | 11.1% |  |  | 4 | 19.0% |  |  | 18 | 56.3% |
| Pharmacist with ID training |  | 27 | 35.1% |  |  | 4 | 22.2% |  |  | 8 | 38.1% |  |  | 10 | 31.3% |
| Pharmacist without or unknown ID training |  | 34 | 44.2% |  |  | 10 | 55.6% |  |  | 7 | 33.3% |  |  | 15 | 46.9% |
| Nurse |  | 40 | 51.9% |  |  | 5 | 27.8% |  |  | 12 | 57.1% |  |  | 20 | 62.5% |
| ASP program measures aggregate antibiotic use | 77 |  | 0.0% |  | 18 |  | 0.0% |  | 21 |  | 0.0% |  | 32 |  | 0.0% |
| in hospital inpatient units |  | 57 | 74.0% |  |  | 16 | 88.9% |  |  | 19 | 90.5% |  |  | 16 | 50.0% |
| in outpatient clinics associated with hospital |  | 17 | 22.1% |  |  | 1 | 5.6% |  |  | 5 | 23.8% |  |  | 9 | 28.1% |
| at the time of discharge from hospital |  | 17 | 22.1% |  |  | 2 | 11.1% |  |  | 5 | 23.8% |  |  | 8 | 25.0% |
| emergency room |  | 16 | 20.8% |  |  | 5 | 27.8% |  |  | 0 | 0.0% |  |  | 10 | 31.3% |
| urgent care facilities associated with hospital |  | 10 | 13.0% |  |  | 3 | 16.7% |  |  | 1 | 4.8% |  |  | 6 | 18.8% |
| nursing homes, long-term care facilities, or rehabilitation facilities associated with hospital |  | 3 | 3.9% |  |  | 0 | 0.0% |  |  | 0 | 0.0% |  |  | 3 | 9.4% |
| ASP program does not measure aggregate antibiotic use |  | 14 | 18.2% |  |  | 2 | 11.1% |  |  | 2 | 9.5% |  |  | 10 | 31.3% |
| Respondent does not know whether aggregate antibiotic use is measured |  | 5 | 6.5% |  |  | 0 | 0.0% |  |  | 0 | 0.0% |  |  | 5 | 15.6% |
| ASP program has interventions in place to improve antibiotic use | 77 |  | 0.0% |  | 18 |  | 0.0% |  | 21 |  | 0.0% |  | 32 |  | 0.0% |
| in hospital inpatient units |  | 60 | 77.9% |  |  | 17 | 94.4% |  |  | 18 | 85.7% |  |  | 19 | 59.4% |
| in outpatient clinics associated with hospital |  | 27 | 35.1% |  |  | 4 | 22.2% |  |  | 4 | 19.0% |  |  | 16 | 50.0% |
| at the time of discharge from hospital |  | 17 | 22.1% |  |  | 5 | 27.8% |  |  | 2 | 9.5% |  |  | 7 | 21.9% |
| emergency room |  | 27 | 35.1% |  |  | 10 | 55.6% |  |  | 2 | 9.5% |  |  | 13 | 40.6% |
| urgent care facilities associated with hospital |  | 10 | 13.0% |  |  | 3 | 16.7% |  |  | 2 | 9.5% |  |  | 4 | 12.5% |
| nursing homes, long-term care facilities, or rehabilitation facilities associated with hospital |  | 3 | 3.9% |  |  | 0 | 0.0% |  |  | 0 | 0.0% |  |  | 3 | 9.4% |
| ASP program does not have interventions in place to improve antibiotic use |  | 11 | 14.3% |  |  | 0 | 0.0% |  |  | 2 | 9.5% |  |  | 9 | 28.1% |
| Respondent does not know if the ASP program has interventions in place to improve antibiotic use |  | 4 | 5.2% |  |  | 0 | 0.0% |  |  | 0 | 0.0% |  |  | 4 | 12.5% |
| Program provides tele-stewardship | 114 | 14 | 12.3% |  | 30 | 6 | 20.0% |  | 25 | 4 | 16.0% |  | 52 | 4 | 7.7% |
| Program receives tele-stewardship | 114 | 12 | 10.5% |  | 30 | 5 | 16.7% |  | 25 | 1 | 4.0% |  | 52 | 6 | 11.5% |
| Diagnostic Stewardship Activities | 112 |  |  |  | 29 |  |  |  | 25 |  | 0.0% |  | 52 |  | 0.0% |
| Program has strategies in place to reduce urine testing |  | 70 | 62.5% |  |  | 13 | 44.8% |  |  | 20 | 80.0% |  |  | 32 | 61.5% |
| Program has strategies in place to reduce blood cultures testing |  | 65 | 58.0% |  |  | 16 | 55.2% |  |  | 18 | 72.0% |  |  | 27 | 51.9% |
| Program has strategies in place to reduce respiratory cultures testing |  | 47 | 42.0% |  |  | 14 | 48.3% |  |  | 12 | 48.0% |  |  | 16 | 30.8% |
| Program has strategies in place to reduce use of respiratory PCR panel |  | 37 | 33.0% |  |  | 8 | 27.6% |  |  | 11 | 44.0% |  |  | 16 | 30.8% |
| Program has strategies in place to reduce use of stool GI pathogen PCR panel |  | 33 | 29.5% |  |  | 7 | 24.1% |  |  | 11 | 44.0% |  |  | 12 | 23.1% |

N represents available answers for each of the questions.

Supplemental Table 3: Covid-19 Response

|  | **Total** | | |  | **Latin America & Caribbean** | | |  | **South Asia** | | |  | **Sub-Saharan Africa** | | |
| --- | --- | --- | --- | --- | --- | --- | --- | --- | --- | --- | --- | --- | --- | --- | --- |
|  | N | n | % |  | N | n | % |  | N | n | % |  | N | n | % |
| Program was very or extremely effective | 102 | 55 | 53.9% |  | 27 | 14 | 51.9% |  | 22 | 15 | 68.2% |  | 46 | 20 | 43.5% |
| Organization the facility relied on for information about COVID-19 during the pandemic | 102 |  |  |  | 27 |  |  |  | 22 |  |  |  | 46 |  |  |
| National Ministry of Health in own country |  | 81 | 79.4% |  |  | 20 | 74.1% |  |  | 18 | 81.8% |  |  | 37 | 80.4% |
| State and/ or local health department |  | 37 | 36.3% |  |  | 7 | 25.9% |  |  | 11 | 50.0% |  |  | 16 | 34.8% |
| A local hospital or local health organization |  | 16 | 15.7% |  |  | 9 | 33.3% |  |  | 2 | 9.1% |  |  | 3 | 6.5% |
| National Infectious Diseases Society in own country |  | 27 | 26.5% |  |  | 9 | 33.3% |  |  | 3 | 13.6% |  |  | 11 | 23.9% |
| National Society of Professionals in IPC in own country |  | 21 | 20.6% |  |  | 3 | 11.1% |  |  | 2 | 9.1% |  |  | 12 | 26.1% |
| United States Centers for Disease Control and Prevention |  | 57 | 55.9% |  |  | 13 | 48.1% |  |  | 9 | 40.9% |  |  | 31 | 67.4% |
| World Health Organization |  | 67 | 65.7% |  |  | 17 | 63.0% |  |  | 12 | 54.5% |  |  | 33 | 71.7% |
| Infectious Diseases Society of America |  | 18 | 17.6% |  |  | 9 | 33.3% |  |  | 5 | 22.7% |  |  | 1 | 2.2% |
| Society for Healthcare Epidemiology of America |  | 18 | 17.6% |  |  | 8 | 29.6% |  |  | 5 | 22.7% |  |  | 1 | 2.2% |
| Facility designated areas to care for COVID-19 patients | 102 | 95 | 93.1% |  | 27 | 24 | 88.9% |  | 22 | 22 | 100.0% |  | 46 | 42 | 91.3% |
| Facility opened new units to care for COVID-19 patients | 102 | 79 | 77.5% |  | 27 | 22 | 81.5% |  | 22 | 17 | 77.3% |  | 46 | 35 | 76.1% |
| Facility experienced staff shortages due to absences and/or illness | 102 | 88 | 86.3% |  | 27 | 24 | 88.9% |  | 22 | 17 | 77.3% |  | 46 | 40 | 87.0% |
| Facility experienced increased loss of staff due to resignations, etc. | 102 | 41 | 40.2% |  | 27 | 16 | 59.3% |  | 22 | 8 | 36.4% |  | 46 | 14 | 30.4% |
| Facility experienced shortage of supplies during the COVID-19 pandemic | 102 |  |  |  | 27 |  |  |  | 22 |  |  |  | 46 |  | 0.0% |
| N95 masks |  | 58 | 56.9% |  |  | 14 | 51.9% |  |  | 5 | 22.7% |  |  | 34 | 73.9% |
| Powered air-purifying respirators |  | 32 | 31.4% |  |  | 2 | 7.4% |  |  | 5 | 22.7% |  |  | 21 | 45.7% |
| Alcohol-based hand sanitizer |  | 46 | 45.1% |  |  | 8 | 29.6% |  |  | 5 | 22.7% |  |  | 31 | 67.4% |
| Gowns |  | 45 | 44.1% |  |  | 9 | 33.3% |  |  | 4 | 18.2% |  |  | 28 | 60.9% |
| Gloves |  | 39 | 38.2% |  |  | 7 | 25.9% |  |  | 4 | 18.2% |  |  | 26 | 56.5% |
| Surgical masks |  | 42 | 41.2% |  |  | 8 | 29.6% |  |  | 3 | 13.6% |  |  | 30 | 65.2% |
| Full face shields |  | 31 | 30.4% |  |  | 2 | 7.4% |  |  | 4 | 18.2% |  |  | 23 | 50.0% |
| Eye shields/ goggles |  | 31 | 30.4% |  |  | 2 | 7.4% |  |  | 4 | 18.2% |  |  | 23 | 50.0% |
| Disinfectant wipes |  | 39 | 38.2% |  |  | 5 | 18.5% |  |  | 4 | 18.2% |  |  | 29 | 63.0% |
| Facility did not experience shortage of supplies during the COVID-19 pandemic |  | 31 | 30.4% |  |  | 8 | 29.6% |  |  | 16 | 72.7% |  |  | 6 | 13.0% |
| Facility experienced moderate or extreme financial hardship | 102 | 58 | 56.9% |  | 27 | 10 | 37.0% |  | 22 | 9 | 40.9% |  | 46 | 34 | 73.9% |
|  |  |  |  |  |  |  |  |  |  |  |  |  |  |  |  |

N represents available answers for each of the questions.

Supplement Table 4: Perceived Resource Needs for Important Infection Prevention and Related Functions

|  | **Total** | | |  | **Latin America & Caribbean** | | |  | **South Asia** | | |  | **Sub-Saharan Africa** | | |
| --- | --- | --- | --- | --- | --- | --- | --- | --- | --- | --- | --- | --- | --- | --- | --- |
|  | N | n | % |  | N | n | % |  | N | n | % |  | N | n | % |
| Preventing CLABSI | 81 |  |  |  | 19 |  |  |  | 18 |  |  |  | 37 |  |  |
| Not applicable: I am not responsible for this activity |  | 4 | 4.9% |  |  | 1 | 5.3% |  |  | 0 | 0.0% |  |  | 3 | 8.1% |
| No additional resources needed |  | 6 | 7.4% |  |  | 2 | 10.5% |  |  | 3 | 16.7% |  |  | 0 | 0.0% |
| I need additional training |  | 17 | 21.0% |  |  | 2 | 10.5% |  |  | 4 | 22.2% |  |  | 11 | 29.7% |
| My team members need additional training |  | 8 | 9.9% |  |  | 3 | 15.8% |  |  | 2 | 11.1% |  |  | 3 | 8.1% |
| I need additional resources to educate frontline clinical personnel |  | 25 | 30.9% |  |  | 4 | 21.1% |  |  | 7 | 38.9% |  |  | 11 | 29.7% |
| I need additional hospital leadership support and engagement |  | 13 | 16.0% |  |  | 4 | 21.1% |  |  | 1 | 5.6% |  |  | 5 | 13.5% |
| I need additional supplies |  | 2 | 2.5% |  |  | 0 | 0.0% |  |  | 0 | 0.0% |  |  | 2 | 5.4% |
| I need additional IPC staff |  | 3 | 3.7% |  |  | 3 | 15.8% |  |  | 0 | 0.0% |  |  | 0 | 0.0% |
| I need additional lab capacity |  | 3 | 3.7% |  |  | 0 | 0.0% |  |  | 1 | 5.6% |  |  | 2 | 5.4% |
|  |  |  |  |  |  |  |  |  |  |  |  |  |  |  |  |
| Preventing SSI | 81 |  |  |  | 19 |  |  |  | 18 |  |  |  | 37 |  |  |
| Not applicable: I am not responsible for this activity |  | 4 | 4.9% |  |  | 1 | 5.3% |  |  | 2 | 11.1% |  |  | 1 | 2.7% |
| No additional resources needed |  | 7 | 8.6% |  |  | 0 | 0.0% |  |  | 5 | 27.8% |  |  | 1 | 2.7% |
| I need additional training |  | 13 | 16.0% |  |  | 2 | 10.5% |  |  | 2 | 11.1% |  |  | 8 | 21.6% |
| My team members need additional training |  | 11 | 13.6% |  |  | 3 | 15.8% |  |  | 3 | 16.7% |  |  | 4 | 10.8% |
| I need additional resources to educate frontline clinical personnel |  | 23 | 28.4% |  |  | 3 | 15.8% |  |  | 4 | 22.2% |  |  | 14 | 37.8% |
| I need additional hospital leadership support and engagement |  | 16 | 19.8% |  |  | 6 | 31.6% |  |  | 2 | 11.1% |  |  | 6 | 16.2% |
| I need additional supplies |  | 2 | 2.5% |  |  | 1 | 5.3% |  |  | 0 | 0.0% |  |  | 1 | 2.7% |
| I need additional IPC staff |  | 2 | 2.5% |  |  | 2 | 10.5% |  |  | 0 | 0.0% |  |  | 0 | 0.0% |
| I need additional lab capacity |  | 3 | 3.7% |  |  | 1 | 5.3% |  |  | 0 | 0.0% |  |  | 2 | 5.4% |
|  |  |  |  |  |  |  |  |  |  |  |  |  |  |  |  |
| Preventing VAE/ VAP | 81 |  |  |  | 19 |  |  |  | 18 |  |  |  | 37 |  |  |
| Not applicable: I am not responsible for this activity |  | 4 | 4.9% |  |  | 1 | 5.3% |  |  | 0 | 0.0% |  |  | 3 | 8.1% |
| No additional resources needed |  | 10 | 12.3% |  |  | 2 | 10.5% |  |  | 4 | 22.2% |  |  | 2 | 5.4% |
| I need additional training |  | 17 | 21.0% |  |  | 3 | 15.8% |  |  | 3 | 16.7% |  |  | 10 | 27.0% |
| My team members need additional training |  | 11 | 13.6% |  |  | 2 | 10.5% |  |  | 4 | 22.2% |  |  | 5 | 13.5% |
| I need additional resources to educate frontline clinical personnel |  | 23 | 28.4% |  |  | 5 | 26.3% |  |  | 5 | 27.8% |  |  | 11 | 29.7% |
| I need additional hospital leadership support and engagement |  | 12 | 14.8% |  |  | 4 | 21.1% |  |  | 2 | 11.1% |  |  | 4 | 10.8% |
| I need additional supplies |  | 2 | 2.5% |  |  | 1 | 5.3% |  |  | 0 | 0.0% |  |  | 1 | 2.7% |
| I need additional IPC staff |  | 1 | 1.2% |  |  | 1 | 5.3% |  |  | 0 | 0.0% |  |  | 0 | 0.0% |
| I need additional lab capacity |  | 1 | 1.2% |  |  | 0 | 0.0% |  |  | 0 | 0.0% |  |  | 1 | 2.7% |
|  |  |  |  |  |  |  |  |  |  |  |  |  |  |  |  |
| Outbreak identification and management | 81 |  |  |  | 19 |  |  |  | 18 |  |  |  | 37 |  |  |
| Not applicable: I am not responsible for this activity |  | 0 | 0.0% |  |  | 0 | 0.0% |  |  | 0 | 0.0% |  |  | 0 | 0.0% |
| No additional resources needed |  | 11 | 13.6% |  |  | 5 | 26.3% |  |  | 2 | 11.1% |  |  | 2 | 5.4% |
| I need additional training |  | 18 | 22.2% |  |  | 2 | 10.5% |  |  | 5 | 27.8% |  |  | 10 | 27.0% |
| My team members need additional training |  | 10 | 12.3% |  |  | 2 | 10.5% |  |  | 3 | 16.7% |  |  | 5 | 13.5% |
| I need additional resources to educate frontline clinical personnel |  | 14 | 17.3% |  |  | 4 | 21.1% |  |  | 4 | 22.2% |  |  | 4 | 10.8% |
| I need additional hospital leadership support and engagement |  | 17 | 21.0% |  |  | 1 | 5.3% |  |  | 2 | 11.1% |  |  | 13 | 35.1% |
| I need additional supplies |  | 1 | 1.2% |  |  | 0 | 0.0% |  |  | 0 | 0.0% |  |  | 1 | 2.7% |
| I need additional IPC staff |  | 5 | 6.2% |  |  | 1 | 5.3% |  |  | 2 | 11.1% |  |  | 1 | 2.7% |
| I need additional lab capacity |  | 5 | 6.2% |  |  | 4 | 21.1% |  |  | 0 | 0.0% |  |  | 1 | 2.7% |
|  |  |  |  |  |  |  |  |  |  |  |  |  |  |  |  |
| Emerging infectious disease preparation and response | 80 |  |  |  | 19 |  |  |  | 18 |  |  |  | 36 |  |  |
| Not applicable: I am not responsible for this activity |  | 0 | 0.0% |  |  | 0 | 0.0% |  |  | 0 | 0.0% |  |  | 0 | 0.0% |
| No additional resources needed |  | 8 | 10.0% |  |  | 2 | 10.5% |  |  | 2 | 11.1% |  |  | 2 | 5.6% |
| I need additional training |  | 15 | 18.8% |  |  | 3 | 15.8% |  |  | 5 | 27.8% |  |  | 7 | 19.4% |
| My team members need additional training |  | 13 | 16.3% |  |  | 3 | 15.8% |  |  | 3 | 16.7% |  |  | 7 | 19.4% |
| I need additional resources to educate frontline clinical personnel |  | 14 | 17.5% |  |  | 1 | 5.3% |  |  | 4 | 22.2% |  |  | 5 | 13.9% |
| I need additional hospital leadership support and engagement |  | 16 | 20.0% |  |  | 3 | 15.8% |  |  | 2 | 11.1% |  |  | 10 | 27.8% |
| I need additional supplies |  | 2 | 2.5% |  |  | 2 | 10.5% |  |  | 0 | 0.0% |  |  | 0 | 0.0% |
| I need additional IPC staff |  | 6 | 7.5% |  |  | 4 | 21.1% |  |  | 1 | 5.6% |  |  | 1 | 2.8% |
| I need additional lab capacity |  | 6 | 7.5% |  |  | 1 | 5.3% |  |  | 1 | 5.6% |  |  | 4 | 11.1% |

N represents available answers for each of the questions.
