# Supplementary material for: Infection prevention and healthcare epidemiology professionals in low- and middle-income countries: a needs assessment survey and call for action
Source: BMJ Glob Health. 2025 Dec 19;10(12):e018265. doi: 10.1136/bmjgh-2024-018265 (PMC12716524; doi:10.1136/bmjgh-2024-018265)
Supplement: online supplemental file 1 [file bmjgh-10-12-s002.pdf]

SHEA
The Society for Healthcare Epidemiology of America

LMIC Survey

About the Survey Responder

Page description:
Definitions to assist you when answering the questions below:

Healthcare Epidemiologist:
MD, PhD, or RN with expertise in healthcare epidemiology and infection control.

Infection Preventionist:
Individual, other than the Healthcare Epidemiologist, responsible for the management and day-to-day activities of the Infection Control Program (with or without Certification in Infection Control).

2
1. **What is your role at the facility?** *

- ○ Healthcare epidemiologist
- ○ Infection preventionist
- ○ Other - please specify

112
2. **Which of the following setting types best describes where you work?** *

- ○ Community-based
- ○ Academic/Teaching Affiliated

113
3. **Which of the following setting classifications best describes where you work?** *

- ○ Primary care only
- ○ Acute care, tertiary hospital
- ○ Long-term care
- ○ Specialty (i.e., cancer, women & children, free-standing children's hospital, psychiatric facility, etc.)
- ○ If none of the above, please specify

11

4. **Which of the following describes your healthcare facility?** *

- Government-owned
- Privately owned, not for profit
- Privately owned, for profit
- Other - please specify

6

5. **Number of inpatient beds:** *

- None
- <200
- 200-500
- 500-1000
- >1000

7

6. **Number of ICU beds:** *

- None
- <10
- 11-20
- 21-30
- 31-40
- >41

8

7. **Does your facility offer any of the following Specialty Services/Care for select populations?***

- Outpatient dialysis
- Blood and marrow transplant
- Solid-organ transplant
- Cardiovascular surgery
- Newborn
- None of the above
- Other - please specify

*

9

8. **Number of outpatient clinic facilities:** *

- 0
- 1-5
- 6-10
- 11-20
- 21 or more

10

9. **Does the facility where you work have an emergency department?***

- Yes
- No

Show/hide trigger exists.

116

10. **Region where your facility is located:**
(Click here to find your region) *

- East Asia & Pacific
- Europe & Central Asia
- Latin America & Caribbean
- Middle East & North Africa
- South Asia
- Sub-Saharan Africa

Hidden unless: #10 Question "**Region where your facility is located:**
(Click here to find your region)" is one of the following answers ("Europe & Central Asia")

12

11. **Country where your facility is located:** *

- Albania
- Armenia
- Azerbaijan
- Belarus
- Bosnia & Herzegovina
- Bulgaria
- Georgia
- Kazakhstan
- Kosovo
- Kyrgyz Rep.
- Moldova
- Montenegro
- North Macedonia
- Romania
- Russian Federation
- Serbia
- Tajikistan
- Turkey
- Turkmenistan
- Ukraine
- Uzbekistan

Hidden unless: #10 Question "**Region where your facility is located:**
(Click here to find your region)" is one of the following answers ("East Asia & Pacific")

117

12. **Country where your facility is located:** *

American Samoa
Cambodia
China
Fiji
Indonesia
Kiribati
Lao PDR
Malaysia
Marshall Islands
Micronesia, Fed. Sts.
Mongolia
Myanmar

Papua New Guinea
Philippines
Samoa
Solomon Islands
Thailand
Timor-Leste
Tonga
Tuvalu
Vanuatu
Vietnam

Hidden unless: #10 Question "**Region where your facility is located:**

(Click here to find your region)' is one of the following answers ("Latin America & Caribbean")

118

13. **Country where your facility is located:** *

- Argentina
- Belize
- Bolivia
- Brazil
- Colombia
- Costa Rica
- Cuba
- Dominica
- Dominican Republic
- Ecuador
- El Salvador
- Grenada
- Guatemala
- Guyana
- Haiti
- Honduras
- Jamaica
- Mexico
- Nicaragua
- Panama
- Paraguay
- Peru
- St. Lucia
- St. Vincent and the Grenadines
- Suriname
- Uruguay
- Venezuela, RB

Hidden unless: #10 Question "**Region where your facility is located:**
(Click here to find your region)" is one of the following answers ("Middle East & North Africa")

119

14. **Country where your facility is located:** *

- Algeria
- Djibouti
- Egypt, Arab Rep.
- Iran, Islamic Rep.
- Iraq
- Jordan
- Lebanon
- Libya
- Morocco
- Syrian Arab Rep.
- Tunisia
- West Bank and Gaza
- Yemen, Rep.

Hidden unless: #10 Question "**Region where your facility is located:** *(Click here to find your region)*" is one of the following answers ("South Asia")

120

15. **Country where your facility is located:** *

Afghanistan
Bangladesh
Bhutan
India
Maldives
Nepal
Pakistan
Sri Lanka

Hidden unless: #10 Question "**Region where your facility is located:**
(Click here to find your region)" is one of the following answers ("Sub-Saharan Africa")

122

16. **Country where your facility is located:** *

Angola
Benin
Botswana
Burkina Faso
Burundi
Cabo Verde
Cameroon
Central African Rep.
Chad
Comoros
Congo, Dem. Rep.
Congo, Rep.
Cote d'Ivoire
Eritrea
Equatorial Guinea
Eswatini
Ethiopia
Gabon
Gambia
Ghana
Guinea
Guinea-Bissau
Kenya
Lesotho
Liberia
Madagasgar
Malawi
Mali
Mauritania
Mayotte
Mozambique
Namibia
Niger
Nigeria
Rwanda
Sao Tome and Principe
Senegal
Sierra Leone
Somalia
South Africa
South Sudan
Sudan
Tanzania
Togo
Tunisia
Uganda
Zambia
Zimbabwe

132

17. **Is there anything else you would like to share about your facility or other facilities in your region?**

About the Program

Show/hide trigger exists.

4

18. **Does the facility where you work have an infection prevention & control (IPC) program?***

- No
- Yes, without clearly defined objectives
- Yes, with clearly defined objectives and annual activity plan

Hidden unless: #18 Question "**Does the facility where you work have an infection prevention & control (IPC) program?** is one of the following answers ("Yes, without clearly defined objectives","Yes, with clearly defined objectives and annual activity plan")

17

19. **Are you in charge of the IPC program at your facility?***

- Yes
- No

Hidden unless: #18 Question "**Does the facility where you work have an infection prevention & control (IPC) program?** is one of the following answers ("Yes, without clearly defined objectives","Yes, with clearly defined objectives and annual activity plan")

18

20. **Is the IPC program supported by an IPC team?***

- Yes
- No
- Not a team, only an IPC focal person

Hidden unless: #18 Question "**Does the facility where you work have an infection prevention & control (IPC) program?** is one of the following answers ("Yes, without clearly defined objectives","Yes, with clearly defined objectives and annual activity plan")

19

21. **Does the IPC team have at least one full-time IPC professional or equivalent (nurse or doctor working 100% in IPC) available?** *

- Yes, one per > 250 beds
- Yes, one per ≤ 250 beds
- No, only a part-time IPC professional available
- No IPC professional available
- I don't know

Hidden unless: (#20 Question "**Is the IPC program supported by an IPC team?**" is one of the following answers ("Yes") AND #18 Question "**Does the facility where you work have an infection prevention & control (IPC) program?**" is one of the following answers ("Yes, without clearly defined objectives","Yes, with clearly defined objectives and annual activity plan"))

21

22. **Does the IPC team include both doctors and nurses?**

- Yes
- No
- I don't know

Hidden unless: #18 Question "**Does the facility where you work have an infection prevention & control (IPC) program?** is one of the following answers ("Yes, without clearly defined objectives","Yes, with clearly defined objectives and annual activity plan")

22

23. **Do you have an IPC committee actively supporting the IPC program?** *

- Yes
- No

24

24. **Are any of the following professional groups actively supporting your job role?** *(select all that apply)* *

- Senior facility leadership (i.e., administrative director, chief executive officer/CEO, medical director)
- Senior clinical staff (i.e., physician, nurse)
- Facility management (i.e., biosafety, waste, those tasked with addressing water, sanitation, & hygiene/WASH)
- No, none of the above

25

25. **Is your job effectiveness measured by clearly defined IPC objectives (that is, in specific critical areas)?** *

- Yes, IPC objectives only
- Yes, IPC objectives and measurable outcome indicators (that is, adequate measures for improvement)
- Yes, IPC objectives, measurable outcome indicators and set future targets
- No

Hidden unless: #18 Question "**Does the facility where you work have an infection prevention & control (IPC) program?** is one of the following answers ("Yes, without clearly defined objectives","Yes, with clearly defined objectives and annual activity plan")

26

26. **Does the senior facility leadership show clear commitment and support for the IPC program by an allocated budget specifically for the IPC program (that is, covering IPC activities, including salaries)?** *

- Yes
- No

Hidden unless: #18 Question "**Does the facility where you work have an infection prevention & control (IPC) program?** is one of the following answers ("Yes, without clearly defined objectives","Yes, with clearly defined objectives and annual activity plan")

114

27. **Does the senior facility leadership show clear commitment and support for the IPC program by demonstrable support for IPC objectives and indicators within the facility (for example, at executive level meetings, executive rounds, participation in morbidity and mortality meetings)?** *

- Yes
- No

Show/hide trigger exists.

27

28. **Does your facility have an antimicrobial stewardship program?** *

- Yes
- No
- I don't know

Hidden unless: #28 Question "**Does your facility have an antimicrobial stewardship program?**" is one of the following answers ("Yes","I don't know")

28

29. **Who is on your antimicrobial stewardship team?** *(select all that apply)* *

- Infection Preventionist
- Infectious Disease Physician
- Hospital Medicine or Internal Medicine Physician
- Other Physician
- Pharmacist (with infectious diseases training)
- Pharmacist (without OR unknown infectious diseases training)
- Nurse
- Other - please specify

*

Hidden unless: #28 Question "**Does your facility have an antimicrobial stewardship program?**" is one of the following answers ("Yes","I don't know")

29

30. **Please indicate whether your antimicrobial stewardship program measures aggregate antibiotic use (i.e., defined daily dose, days of therapy) for use in any of the following settings:** *(select all that apply)* *

- ☐ Inpatient units (in hospital)
- ☐ Outpatient/ambulatory clinics associated with your hospital
- ☐ At the time of discharge from the hospital
- ☐ Emergency room(s)
- ☐ Urgent care facilities associated with your hospital
- ☐ Nursing homes, long-term care facilities, or rehabilitation facilities associated with your hospital
- ☐ None of the above
- ☐ I don't know

Hidden unless: #28 Question "**Does your facility have an antimicrobial stewardship program?**" is one of the following answers ("Yes","I don't know")

30

31. **Please indicate whether your antimicrobial stewardship program has one or more interventions in place to improve appropriate antibiotic use in any of the following settings:** *(select all that apply)* *

- ☐ Inpatient units (in hospital)
- ☐ Outpatient/ambulatory clinics associated with your hospital
- ☐ At the time of discharge from the hospital
- ☐ Emergency room(s)
- ☐ Urgent care facilities associated with your hospital
- ☐ Nursing homes, long-term care facilities, or rehabilitation facilities associated with your hospital
- ☐ None of the above
- ☐ I don't know

31

32. **Does your facility *provide* tele-stewardship (i.e., using remote monitoring to help other facilities)?** *

- ○ Yes
- ○ No
- ○ I don't know
- ○ There are no other facilities that require help with stewardship

32

33. **Does your facility *receive* tele-stewardship (i.e., another group helps you monitor antibiotic use)?** *

- ○ Yes
- ○ No
- ○ I don't know

33

34. **Does your facility have any strategies in place to reduce inappropriate testing for any of the following?***(select all that apply)* *

- ☐ Urine testing (including urine cultures or urinalyses)
- ☐ Blood cultures
- ☐ Respiratory cultures
- ☐ Respiratory panel by PCR
- ☐ Stool gastrointestinal pathogen panel by PCR
- ☐ Other - please specify

*

127

35. **Is there anything else you would like to share about your program or other programs in your region?**

COVID-19 Response

Page description:
The following questions pertain to your facility's experiences during the COVID-19 pandemic at any point from March 2020 until the present.

147

36. **In your opinion, how effective has your facility's pandemic response plan been in addressing COVID-19?** *

- ○ Minimally effective
- ○ Moderately effective
- ○ Very effective
- ○ Extremely effective
- ○ Not applicable, our hospital did not have a pandemic response plan

148

37. **Which organization has your facility relied on for information about COVID-19?** (*select all that apply*) *

- ☐ National Ministry of Health (in the country where you work)
- ☐ State and/or local health department
- ☐ A local hospital or local health organization
- ☐ National Infectious Disease Society (ID society from your own country)
- ☐ National Society of Professionals in Infection Prevention and Epidemiology (society from your own country)
- ☐ Centers for Disease Control and Prevention (CDC)
- ☐ World Health Organization (WHO)
- ☐ Infectious Diseases Society of America (IDSA)
- ☐ Society for Healthcare Epidemiology of America (SHEA)
- ☐ Other - please specify *

149

38. **Has your facility designated areas to care for COVID-19 patients that are separated from non-COVID patients?** *

- ○ Yes, facility wide
- ○ Yes, but only in some units (i.e., ICU)
- ○ No
- ○ I don't know

150

39. **Has your facility opened new units to care for COVID-19 patients?** *

- ○ Yes
- ○ No
- ○ I don't know

151

40. **Has your facility experienced staff shortages due to absences and/or illness during the COVID-19 pandemic?** *

- ○ Yes
- ○ No
- ○ I don't know

152

41. **Has your facility experienced an increased loss of staff (i.e., resignations) in the midst of COVID-19?** *

- ○ Yes
- ○ No
- ○ I don't know

153

42. **Has your facility experienced a shortage of any supplies during the COVID-19 pandemic?** *(select all that apply)* *

- N95 masks
- Powered air-purifying respirators (PAPRs)
- Alcohol-based hand sanitizer
- Gowns
- Gloves
- Surgical masks
- Full face shields
- Eye shields/goggles
- Disinfectant wipes
- No supply shortages experienced
- Other - please specify

*

154

43. **Has your facility experienced financial hardship resulting from the COVID-19 pandemic?** *

- No financial hardship
- Mild financial hardship
- Moderate financial hardship
- Extreme financial hardship
- I don't know

134

44. **Is there anything else you would like to share about COVID-19 response in your facility or in the facilities of colleagues in your region?**

Job Responsibilities; Training; Professional Development

62

45. **What is your professional background training?** *(select all that apply)* *

- Nursing
- Clinical medicine
- Microbiology
- Public health
- Healthcare administration
- Other - please specify

*

50

46. **What are your job responsibilities?** *(select all that apply)*

	Check this box if this is a responsibility of yours	Check this box if you received formal training for this responsibility
Provide education to staff	☐	☐
Develop and implement policies and procedures	☐	☐
Conduct surveillance per regulatory requirement	☐	☐
Compile and report infectious diseases to local public health authorities	☐	☐
Conduct surveillance to promptly identify and interrupt clusters and outbreaks	☐	☐
Serve as liaison between external public health agencies and the hospital	☐	☐
Serve as content expert to troubleshoot infection control related questions and concerns	☐	☐
Occupational Health	☐	☐
Antimicrobial Stewardship	☐	☐
Emerging infectious disease and bioterrisom preparedness and response	☐	☐
Implement bundles of care to reduce healthcare-associated infections	☐	☐
Enter another option	☐	☐
Enter another option	☐	☐
Enter another option	☐	☐

63

47. **What formal training have you received for your job role in infection prevention?** *(select all that apply)* *

- ☐ No formal training
- ☐ WHO training
- ☐ European society training
- ☐ U.S. SHEA training
- ☐ U.S. APIC training
- ☐ Training by the local public health authorities/ministries of health/health departments
- ☐ Infection control training offered by local organizations or training programs
- ☐ Other - please specify

*

64

48. **Have you received any formal training in antimicrobial stewardship?** *

- ○ Yes
- ○ No

65

49. **Have you received any formal training in quality improvement and patient safety tools?** *

- Yes
- No

66

50. **Have you received any formal leadership training?** *

- Yes
- No

67

51. **Do you have paid leave and sponsorship from your facility to participate in at least one conference or meeting per year to refresh/ update your job-related knowledge and skills?** *

- Paid leave only
- Sponsorship only
- Paid leave and sponsorship
- Neither paid leave nor sponsorship

Show/hide trigger exists.

68

52. **Does your facility provide you with technology (i.e., a computer, tablets) to conduct your IPC or antimicrobial stewardship activities (i.e., perform surveillance)?** *

- Yes
- No

Hidden unless: #52 Question "**Does your facility provide you with technology (i.e., a computer, tablets) to conduct your IPC or antimicrobial stewardship activities (i.e., perform surveillance)?** " is one of the following answers ("No")

69

53. **What tools do you use to perform your routine activities at your job?***

- Paper
- Personal cell phone
- Other - please specify

Show/hide trigger exists.

70

54. **Do you have free Wi-Fi access at your facility to help you perform your IPC or antimicrobial stewardship activities (i.e., perform surveillance, read updated guidance)?** *

- Yes
- No

Hidden unless: #54 Question "**Do you have free Wi-Fi access at your facility to help you perform your IPC or antimicrobial stewardship activities (i.e., perform surveillance, read updated guidance)?** " is one of the following answers ("No")

71

55. **Do you use your own Wi-Fi to perform any of your work activities?** *

- Yes
- No

123

56. **Do you experience any challenge besides accessing resources?** *

- No
- Yes - please specify

128

57. **Is there anything else you would like to share about your job responsibilities, training or professional development or those of colleagues in your region?**

Workload & Work Environment

98

58. **The following pertain only to the person completing this survey. For each of the statements below, please indicate the extent of your agreement or disagreement by checking the appropriate box.** *

	Strongly disagree	Disagree	Neither disagree or agree	Agree	Strongly agree
I feel burned out from my work. *					
I have become more callous towards people since I took my job.*					
Spiritual well-being is important for one’s emotional well-being. *					
Religious or spiritual beliefs act as a source of comfort and strength during life’s ups and downs. *					
An organized religious or spiritual community is important to me.*					
Individual self-care practices (i.e., meditation, yoga, listening to music, exercising, communing with nature) is important to me. *					

102

59. **The following pertain to your views of the healthcare facility where you work. For each of the statements below, please indicate the extent of your agreement or disagreement by checking the appropriate box.** *

	Strongly disagree	Disagree	Neither disagree or agree	Agree	Strongly agree
I assert my views on important issues, even though my supervisor may disagree.*	○	○	○	○	○
I personally feel comfortable speaking up when I see a physician not clean his or her hands. *	○	○	○	○	○
When a medical error occurs at this facility, employees are encouraged to discuss mistakes in order to learn how to prevent similar future errors. *	○	○	○	○	○
Leadership is driving us to be a safety-centered institution. *	○	○	○	○	○
I would feel safe being treated here as a patient. *	○	○	○	○	○
If you make a mistake at this facility, it is often held against you.*	○	○	○	○	○
Employees at this facility are able to bring up problems and tough issues.*	○	○	○	○	○
It is safe to try something new at this facility.*	○	○	○	○	○
At this facility, people are too busy to invest time in improvement.*	○	○	○	○	○
In this facility, employees are expected to question leadership.*	○	○	○	○	○
In this facility, authority is concentrated at the top.*	○	○	○	○	○

90

60. **Do you feel you are under any pressure to NOT report healthcare-associated infections at your facility?** *

Always	Sometimes	Rarely	Never
○	○	○	○

105

61. **Among all patient safety issues, how important is hand hygiene at your facility?**

*

Minimally important	Moderately important	Very important	Extremely important
○	○	○	○

106

62. **Please indicate how much you agree or disagree with the following statement: “I feel safe carrying out my work role during the COVID-19 pandemic”.**

*

Strongly disagree	Disagree	Neither disagree or agree	Agree	Strongly agree
○	○	○	○	○

129

63. **Is there anything else you would like to share about your workload or work environment, or those of colleagues in your region?**

Needs & Priorities

107

64. **Please provide your assessment of the necessary resources you would need in order to successfully carry out the follow responsibilities.**

I need... *

	N/A: I am not responsible for this activity	No additional resources needed	additional training	My team members need additional training	additional resources to educate frontline clinical personnel	additional hospital leadership support & engagement	additional supplies	additional IPC staff	additional lab capacity	I need other additional resou *(please speci*
Hand hygiene										
Preventing central-line associated bloodstream infections (CLABSIs)										
Preventing surgical site infections (SSIs)										
Preventing ventilator-associated events/ventilator-associated pneumonia (VAE/VAPs)										
Preventing multidrug-resistant organisms (MDROs)										
Improving antibiotic use										
Outbreak identification & management										
Emerging infectious disease preparation & response										

Min. answers = 3 *(if answered)* Max. answers = 3 *(if answered)*
111

65. **Of the following infection prevention activities, please select and rank the top three (3) priorities for the facilities where you work, with the highest priority being at the top.** *

Drag items from the left-hand list into the right-hand list to order them.

- Hand hygiene
- Preventing CLABSI
- Preventing SSIs
- Preventing VAE/VAPs
- Preventing MDROs
- Improving antibiotic use
- Outbreak identification & management
- Emerging infectious disease preparation & response

130
66. **Is there anything else you would like to share about your program needs and priorities or those of colleagues in your region?**

(untitled)

124
67. **Are you a SHEA member at the time of completing this survey?** *

- Yes
- No

Show/hide trigger exists.
125
68. **Did you use a language translation app in your internet browser to assist with completing this survey?** *

- Yes
- No

Hidden unless: #68 Question "**Did you use a language translation app in your internet browser to assist with completing this survey?** is one of the following answers ("Yes")

126

69. **What language did you complete the survey in?**

*

131

70. **Would you be interested in volunteering for an in-depth interview regarding your responses?**

- No
- Yes (*please provide your name and email address in the text box below so we can contact you. Please note that we may not be able to interview all individuals who volunteer*)

Comments

1

Thank you for taking time to complete our survey. Your input is highly valuable and much appreciated.

Please email Lorenzo Brady at lbrady@shea-online.org if you have any questions or concerns.
